# Drug resistance related genes in lung adenocarcinoma predict patient prognosis and influence the tumor microenvironment

**DOI:** 10.1038/s41598-023-35743-y

**Published:** 2023-06-15

**Authors:** Hui Yu, Wenting Zhang, Xian Rong Xu, Shengjie Chen

**Affiliations:** 1 Department of Thoracic Surgery, Affiliated Hospital of Jiangsu University, No. 438 Jiefang Road, Zhenjiang, 212001 Jiangsu People’s Republic of China; 2 Department of Galactophore, Danyang Maternal and Child Health Hospital, Danyang, 212300 Jiangsu People’s Republic of China

**Keywords:** Biochemistry, Biological techniques, Biophysics, Biotechnology, Cancer, Cell biology, Chemical biology, Computational biology and bioinformatics, Developmental biology, Drug discovery, Genetics, Immunology, Molecular biology, Biogeochemistry, Biomarkers, Diseases, Health care, Medical research, Molecular medicine, Oncology, Pathogenesis, Risk factors, Engineering

## Abstract

Lung adenocarcinoma (LUAD) is the predominant type of non-small lung cancer (NSCLC) with strong invasive ability and poor prognosis. The drug resistance related genes are potentially associated with prognosis of LUAD. Our research aimed to identify the drug resistance related genes and explore their potential prognostic value in LUAD patients. The data used in this study were obtained from The Cancer Genome Atlas (TCGA) and Gene Expression Omnibus (GEO) databases. Firstly, we screened drug resistance related genes in LUAD by differential gene analysis, univariate Cox regression and drug sensitivity analyses. Subsequently, we constructed a risk score model using LASSO Cox regression analysis, and verified whether the risk score can predict the survival of LUAD patients independent of other factors. Moreover, we explored the immune infiltration of 22 immune cells between high-risk and low-risk patients. Totally 10 drug-resistance positively related genes (PLEK2, TFAP2A, KIF20A, S100P, GDF15, HSPB8, SASH1, WASF3, LAMA3 and TCN1) were identified in LUAD. The risk score model of LUAD constructed with these 10 genes could reliably predict the prognosis of LUAD patients. 18 pathways were significantly activated in high-risk group compared with low-risk group. In addition, the infiltration proportion of multiple immune cells was significantly different between high-risk and low-risk groups, and the proportion of M1 phagocytes was significantly higher in the high-risk group compared with the low-risk group. The drug resistance related genes (PLEK2, TFAP2A, KIF20A, S100P, GDF15, HSPB8, SASH1, WASF3, LAMA3 and TCN1) could predict the prognosis of LUAD patients. Clarifying the roles and mechanisms of these 10 genes in regulating drug resistance in LUAD will help to improve individualized clinical treatment protocols and predict patient sensitivity to treatment.

## Introduction

Lung cancer is one of the most severe public health problems worldwide, among which lung adenocarcinoma (LUAD) accounts for 60% of non-small-cell lung cancer (NSCLC)^1^. LUAD is prone to metastasize at an early stage. Thus, the prognosis of LUAD is usually poor and the average 5-year survival rate is less than 20%^2^. In addition, the largest proportion of disability structures caused by lung cancer are adenocarcinomas, squamous cell carcinomas and small cell carcinomas^3^. In recent years, the targeted therapy of LUAD has made great progress but its 5-year overall survival rate remains unsatisfactory because of drug resistance^4^, metastasis^5^ and proliferation^6^. In addition, LUAD cells are able to rapidly acquire drug resistance after initial treatment and usually cannot be treated with chemotherapeutic agents^7,8^. Therefore, exploring determinants of drug resistance in LUAD is necessary to improve clinical efficacy of drugs.

The factors contributing to resistance to therapy include genetic, non-genetic, and external microenvironmental factors^9,10^. Several researchers have reported that the gene mutations, amplifications and deletions in cancer cells could render target protein mutations that are unable to bind drugs and activation of downstream effectors of signaling. Thus, tumor cells are able to escape the effects of therapy (radiotherapy and chemotherapy, targeted therapy and immunotherapy) and continue to proliferate and invade^11^. For instance, the overexpression of DSG2 can promote cell proliferation and migration in LUAD and increase the drug resistance of osimertinib (EGFR tyrosine kinase inhibitor), whereas loss of DSG2 could reverse these phenomena by affecting the signal transduction of EGFR^12^. In addition to these genetic modifications, the external factors in tumor microenvironment (TME) play an important role in promoting tumor progression and drug resistance^13^. Tumor-associated macrophages (TAMs) is one of the crucial cells in the regulation of TME^14^, including the M1 macrophages (restrain tumor progression) and M2 macrophages (promote tumor progression)^15,16^. Lriki et al. have found that TAMs are able to activate STAT3 by secreting IL-6 in small cell lung cancer (SCLC) cells, which increase drug resistance, proliferation and invasion of tumor cells^17^. In lung cancer, the drug resistance-related genes (*DLGAP1*, *SEC14L5, CCDC73*) expression are associated with the infiltration of dendritic cells, macrophages, neutrophils, B cells, CD4+ T cells and CD8+ T cells^18^. These evidences suggested that drug resistance related genes might interact with TME and influence patient response to therapy, in turn affecting the prognosis of LUAD patients. Therefore, exploring drug resistance related genes and their effects on the TAMs will contribute to further understanding the chemo-resistance mechanisms of cancer cells, and predict the prognosis of LUAD patients. However, in the field of LUAD, the drug-related genes and their correlation with infiltration of immune cells have not been comprehensively studied.

Therefore, in this work, we aimed to explore the drug resistance related genes in LUAD patients and analyze their influence on the TME. Our study will provide more information for understanding the link between the drug resistance genes and TME in LUAD patients.

## Materials and methods

### Data sources

A total of 585 LUAD samples were downloaded from The Cancer Genome Atlas (TCGA) database (https://xenabrowser.net/datapages/), and the survival information was shown in Table S1. The GSE43458 (including 80 cancer samples and 30 paracancerous samples), GSE32863 (including 58 cancer samples and 58 paracancerous samples) and GSE42127 (including survival information) datasets were downloaded from the Gene Expression Omnibus (GEO) database (https://www.ncbi.nlm.nih.gov/geo/).

### Differential gene and crossover analysis

The differential gene analysis between cancer samples and paracancerous samples in TCGA-LUAD, GSE43458 and GSE32863 datasets was performed using the limma package of R language (version 4.2.0). The |log2FC| > 1 and p < 0.05 were identified as criteria for screening differentially expressed genes (DEGs). The overlapping genes among the three datasets were obtained by crossover analysis.

### Univariate Cox regression and drug sensitivity analysis

Univariate Cox regression analysis of overlapping genes was performed using the R package “survival” in the TCGA-LUAD dataset. The DEGs were further screened through p < 0.05. Subsequently, drug sensitivity analysis was performed in the GSCA database (http://bioinfo.life.hust.edu.cn/GSCA/#/drug) to screen candidate genes.

### Least absolute shrinkage selection operator (LASSO) Cox regression analysis

The lambda value was calculated by LASSO Cox regression using the glmnet package in R language (version 4.2.0) to further screen prognosis-related genes in LUAD. The lambda is also called the model coefficient ratio. Along with the λ increase, the regression coefficient of each variable β decrease. Some will change to 0, indicating that the variable contributes marginally to the model and can be dropped. The risk score of each sample in TCGA-LUAD dataset was calculated using the candidate genes through the following formula.

$$Risk\, score=\sum _{{\rm{i}}=1}^{\rm{n}}{\rm{Coef}}_{\rm{i}} \times {\rm{X}}_{\rm{i}}$$

Coefi is the risk coefficient of each factor calculated by the LASSO Cox model, Xi is the expression value of each factor, corresponding to the value of mRNA expression.

The values of risk score were determined using R packages survival, survminer and two-sided log rank tests, and the patients were divided into low-risk and high-risk groups according to the median value. Subsequently, the multivariate Cox regression analysis, including gender, cancer stage and risk score, was performed to verify whether the risk score can predict the survival of LUAD patients independent of other factors.

### Mutation analysis

The variant frequency and type of genes were analyzed in the GSCA database to explore the mutations and copy number variation (CNV) of candidate genes.

### Gene set enrichment analysis (GSEA)

Based on the median risk score, samples with a risk score above the median were classified as the high-risk group, and samples below or equal to the median were classified as the low-risk group in the TCGA-LUAD dataset. The DEGs between high-risk and low-risk groups were then subjected to GSEA analysis using the R language (version 4.2.0) function packages Reactomepa and Clusterprofiler.

### Immune cell infiltration analysis

The immune infiltration of 22 immune cells between high-risk and low-risk patients in the TCGA-LUAD dataset was calculated using CIBERSORT, and analyze the correlation between risk score and proportion of immune cell infiltration. The CIBERSORT software can characterize the composition of immune infiltrating cells using preset 547 barcode genes according to the deconvolution algorithm, based on the gene expression matrix. The sum of all estimated proportions of immune cell types in each sample was equal to 1.

## Results

### Differential expression of genes between LUAD and paracancerous tissues

**Figure 1:**
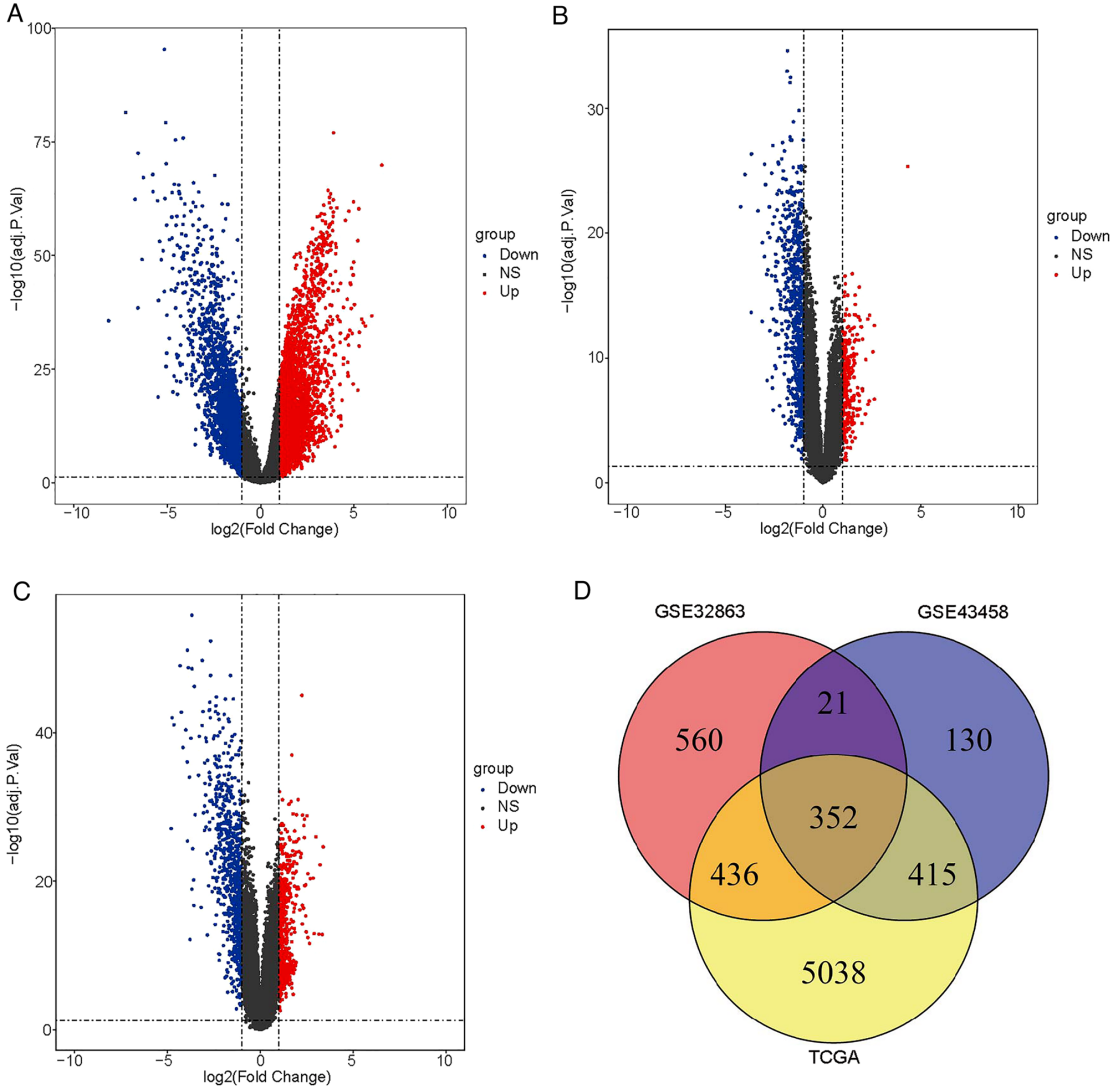
The DEGs between cancer group and paracancerous group in LUAD patients. The volcano plot of DEGs between cancer group and paracancerous sample in TCGA-LUAD dataset (**A**), GSE43458 dataset (**B**) and GSE32863 dataset (**C**). (**D**) The overlapping genes among the three datasets.

Firstly, we analyzed the DEGs between cancer and paracancerous groups in the three datasets (TCGA-LUAD, GSE43458, GSE32863). A total of 6241 DEGs were identified in the cancer group compared with the paracancerous group in TCGA-LUAD dataset, including 4108 upregulated genes and 2133 downregulated genes (Fig. 1A, Fig. S1A). In the GSE43458 dataset, a total of 918 DEGs were obtained in the cancer group compared with the paracancerous group, including 265 upregulated genes and 653 downregulated genes (Fig. 1B, Fig. S1B). From the GSE32863 dataset, a total of 1369 DEGs were screened in the cancer group compared with the paracancerous group, including 583 upregulated genes and 786 downregulated genes (Fig. 1C, Fig. S1C). In addition, we analyzed the overlapping genes among three datasets using crossover analysis, and found that a total of 352 overlapping genes were obtained among the three datasets (Fig. 1D).

### Drug resistance-related genes in LUAD patients

**Figure 2:**
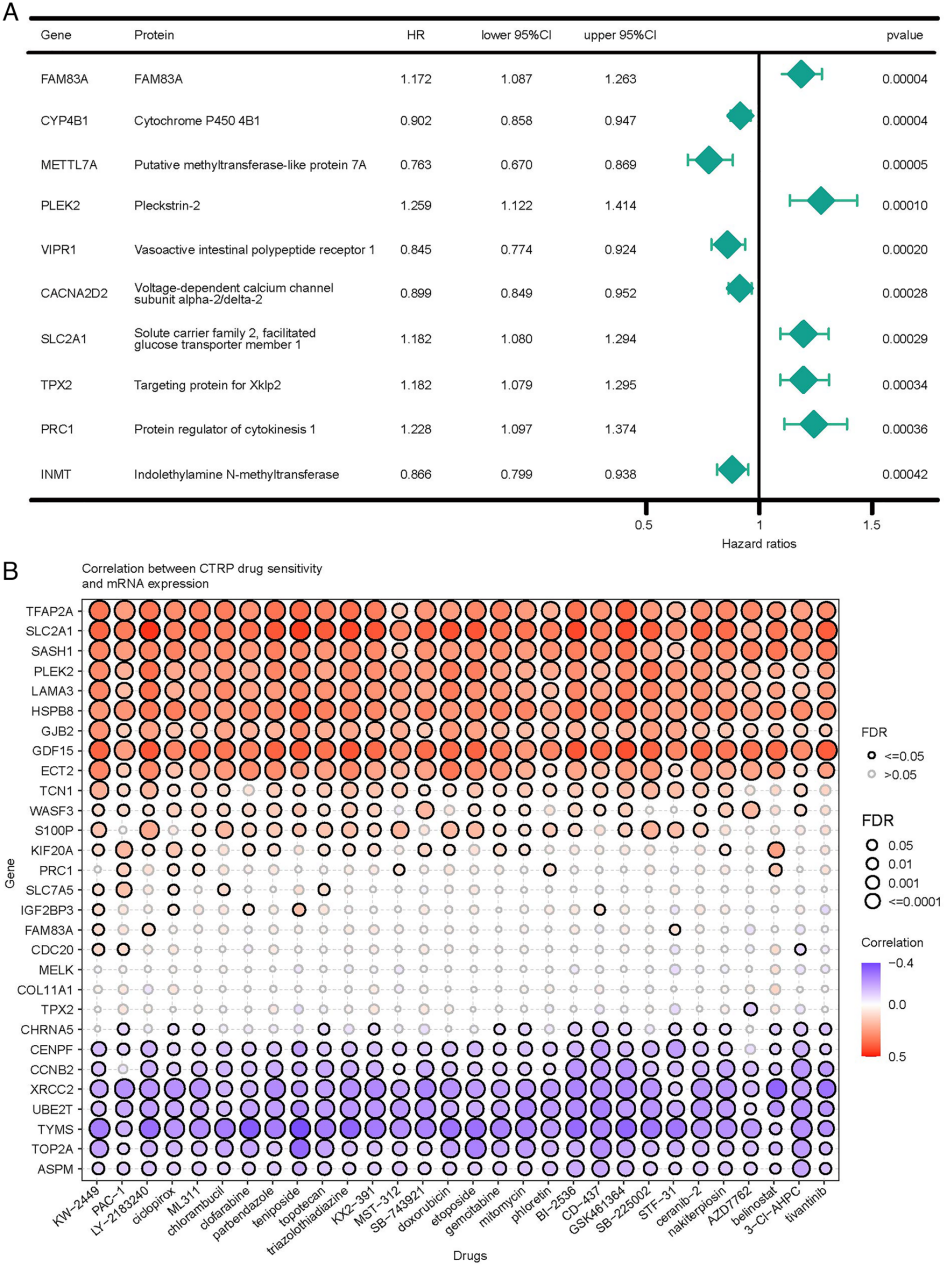
Drug resistance-related genes in LUAD patients. (**A**) Forest plot of univariate Cox regression analysis. (**B**) The map of correlation analysis between candidate and drug resistance related genes.

Next, we analyzed the association between 352 overlapping genes and the prognosis of LUAD using univariate Cox regression analysis. A total of 126 genes were associated with the prognosis of LUAD patients (Table S2). The top ten significantly associated genes were presented in Fig. 2A. Moreover, we analyzed the drug sensitivity of 126 genes and screened the top 13 genes that were positively associated with drug resistance (Fig. 2B).

### Predictive prognostic model for LUAD

**Figure 3:**
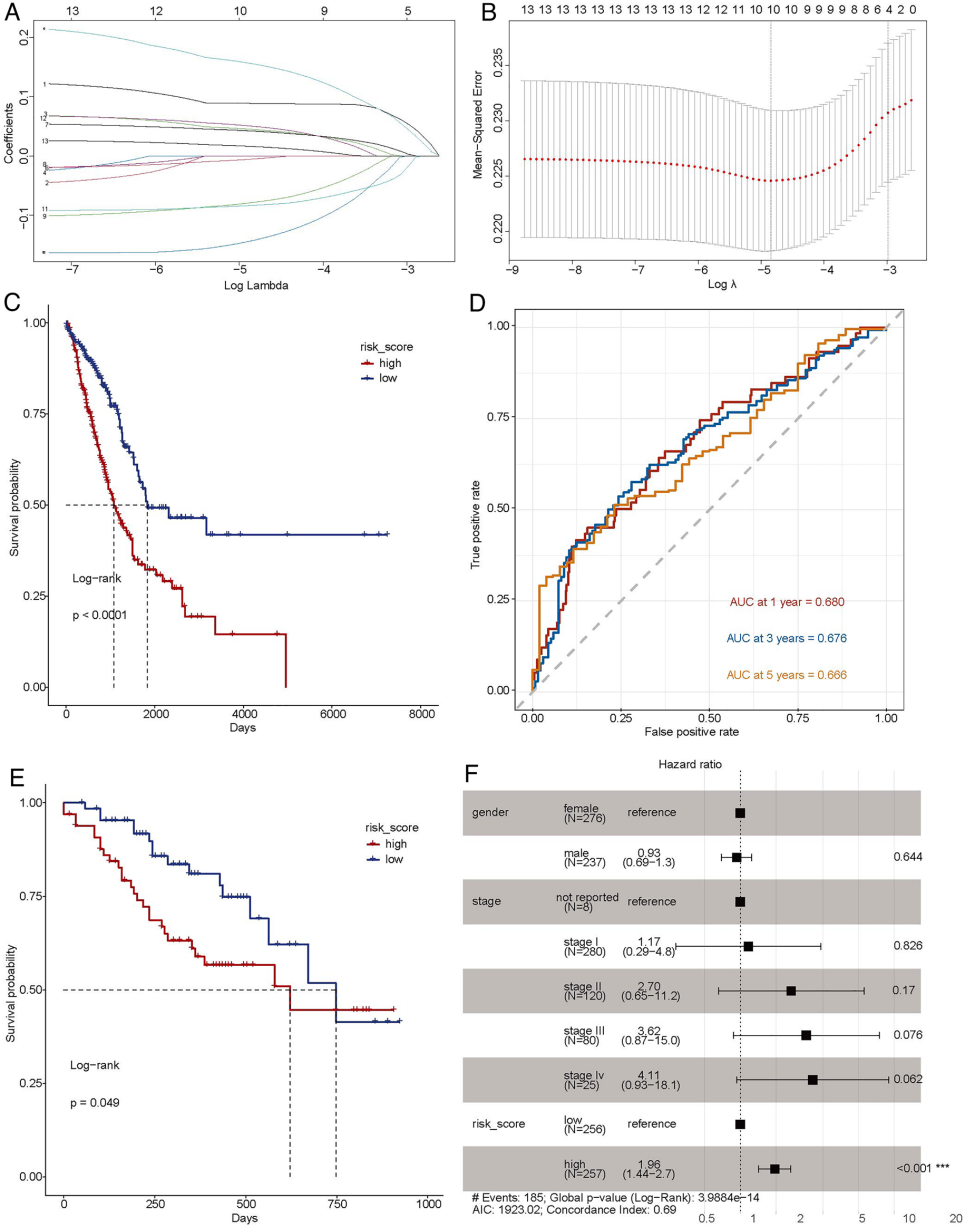
Predictive prognostic model for LUAD. (**A**,**B**) The lambdas quality control plots of LASSO Cox regression analysis. (**C**) Kaplan–Meier curves of high-risk and low-risk groups in TCGA-LUAD dataset. (**D**) The receiver operating characteristic (ROC) curves of the risk score of patients in the TCGA-LUAD dataset, *AUC* area under curve. (**E**) Kaplan–Meier curves of high-risk and low-risk groups in GSE42127 dataset. (**F**) Forest plot of multivariate Cox regression in TCGA-LUAD dataset.

The top 13 genes were subjected to LASSO Cox regression in TCGA-LUAD dataset. The optimal 10 genes (PLEK2, TFAP2A, KIF20A, S100P, GDF15, HSPB8, SASH1, WASF3, LAMA3 and TCN1) were obtained using the value of lambda in LASSO Cox regression analysis (Fig. 3A,B). Subsequently, the expression of genes was weighted with regression coefficient of LASSO Cox regression analysis to construct predictive a prognostic risk score model: Risk score = Expression of PLEK2 × (0.0197135398) + Expression of TFAP2A × (0.0098853327) + Expression of KIF20A × (0.0321060109) + Expression of S100P × (0.0086876008) + Expression of GDF15 × (− 0.0008813305) + Expression of HSPB8 × (− 0.0173687855) + Expression of SASH1 × (− 0.0303346357) + Expression of WASF3 × (− 0.0183514611) + Expression of LAMA3 × (0.0118691125) + Expression of TCN1 × (0.003111329). The patients were divided into high-risk and low-risk groups according to the median risk score. We firstly analyzed the correlation between risk score and prognosis of LUAD patients, and found that the high-risk group had a worse prognosis compared with the low-risk group (Fig. 3C). Moreover, the survival area under curves (AUCs) of 1-, 3-, and 5-year overall survival in TCGA-LUAD dataset were 0.680, 0.676 and 0.666, respectively (Fig. 3D). Next, we multiplied the expression of each gene in GSE42127 dataset with the corresponding coefficient, and then summed the results, resulting in a risk score for each sample as well. All samples in GSE42127 were also grouped according to their risk score median, and then we performed survival analysis and plotted KM curves. Similarly, compared with low-risk patients, the high-risk patients exhibited poorer prognoses (Fig. 3E).

To explore whether the risk score is an independent prognostic indicator, we conducted multivariate Cox regression analysis including the gender, cancer stage and risk score. The risk score was significantly associated with the overall survival of LUAD patients (Fig. 3F), and the high-risk group was associated with poor prognosis compared with the low-risk group (p < 0.001). These results indicated that the risk score model could reliably predict the prognosis of LUAD patients.

### Mutation analysis of 10 crucial genes

**Figure 4:**
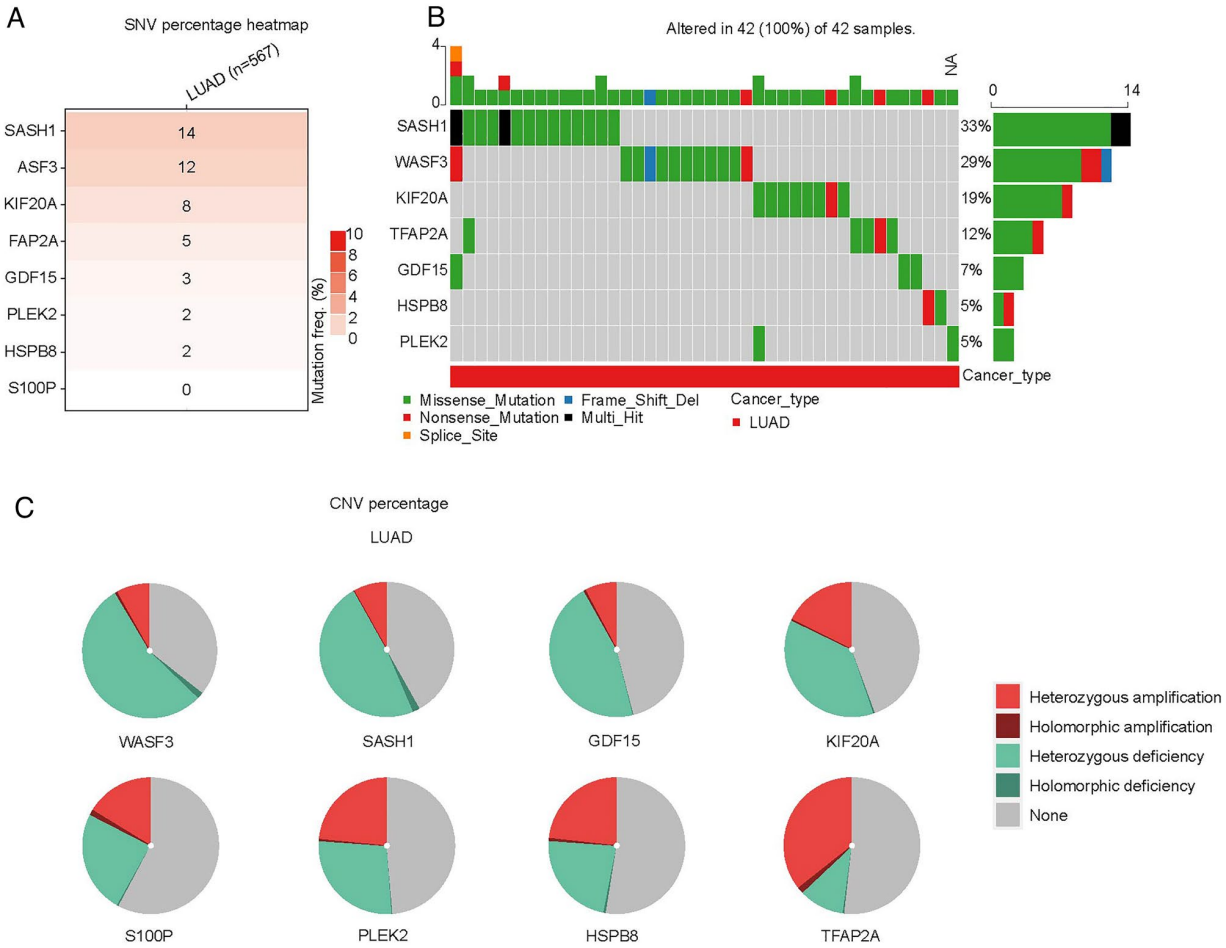
Variant frequency and mutation type of 10 genes in LUAD. (**A**) The mutation rate of genes. (**B**) The mutation type of genes. (**C**) The copy number variation (CNV) types of genes.

In addition, we analyzed the mutation frequency and mutation type of 10 genes in GSCA database, and we found that the frequently mutated gene was SASH1 (14%), followed by WASF3 (12%) (Fig. 4A), and the missense mutations of SASH1 and WASF3 were 33% and 29%, respectively (Fig. 4B). Moreover, we analyzed the CNV of 10 genes and found that the proportion of heterozygous deletions of SASH1 and WASF3 was also highest (Fig. 4C, Table S3).

### Potential pathways between high and low risk patient

**Figure 5:**
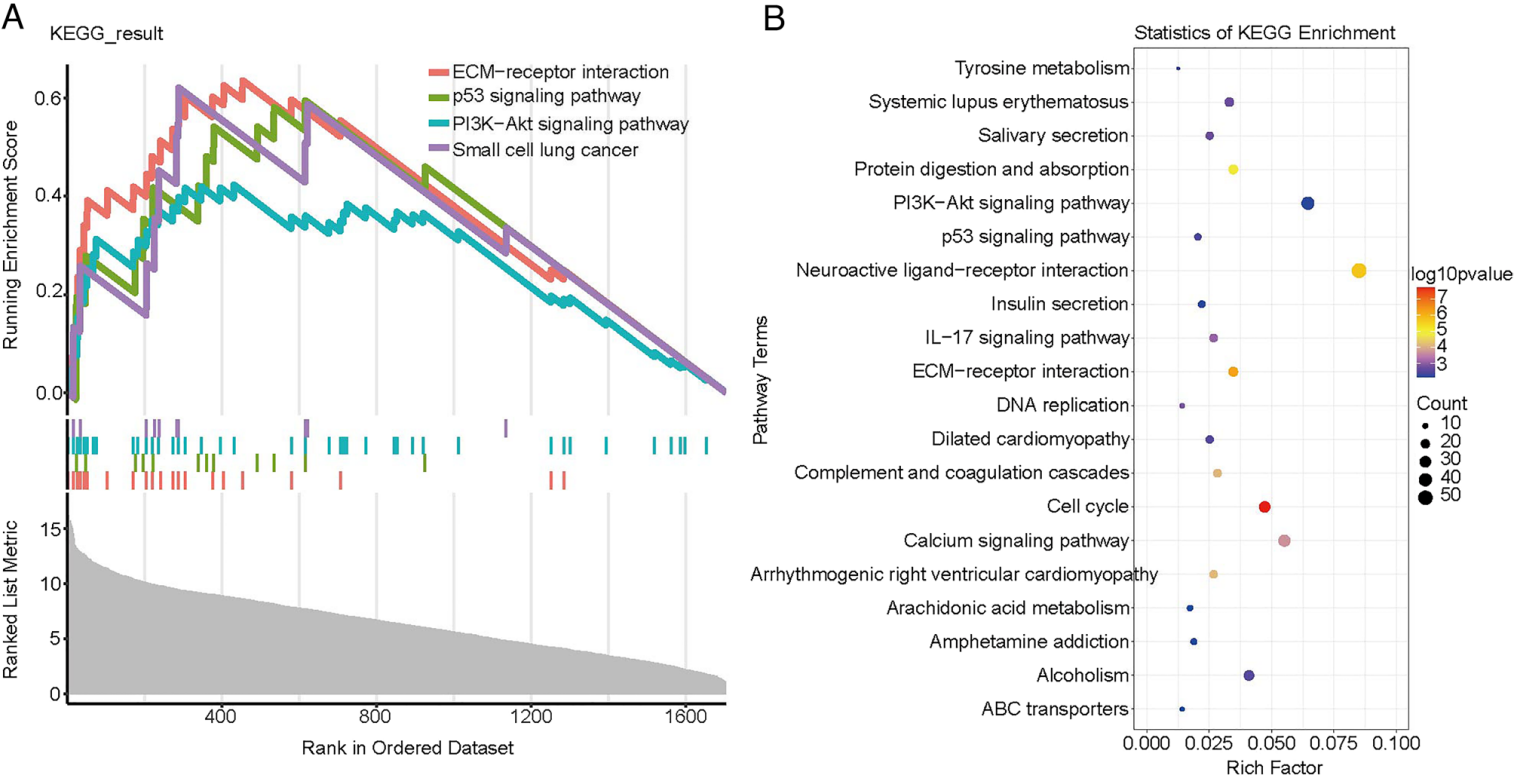
Potential pathways of DEGs between high and low risk patients. (**A**) The result of GSEA analysis. (**B**) The top twenty significantly enriched in KEGG pathways.

Afterwards, we analyzed the DEGs between high-risk group and low-risk group to explore more functional information. GSEA showed that a total of 18 pathways were significantly activated in high-risk group compared with low-risk group, such as extracellular matrix (ECM)-receptor interaction, PI3K-Akt signaling pathway, p53 signaling pathway and small cell lung cancer (Fig. 5A, Table S4). In addition, several similar pathways were also significantly enriched via KEGG enrichment analysis, such as (ECM)-receptor interaction, PI3K-Akt signaling pathway and p53 signaling pathway (Fig. 5B)^19–21^.

### Immune cell infiltration in high-risk and low-risk LUAD patients

**Figure 6:**
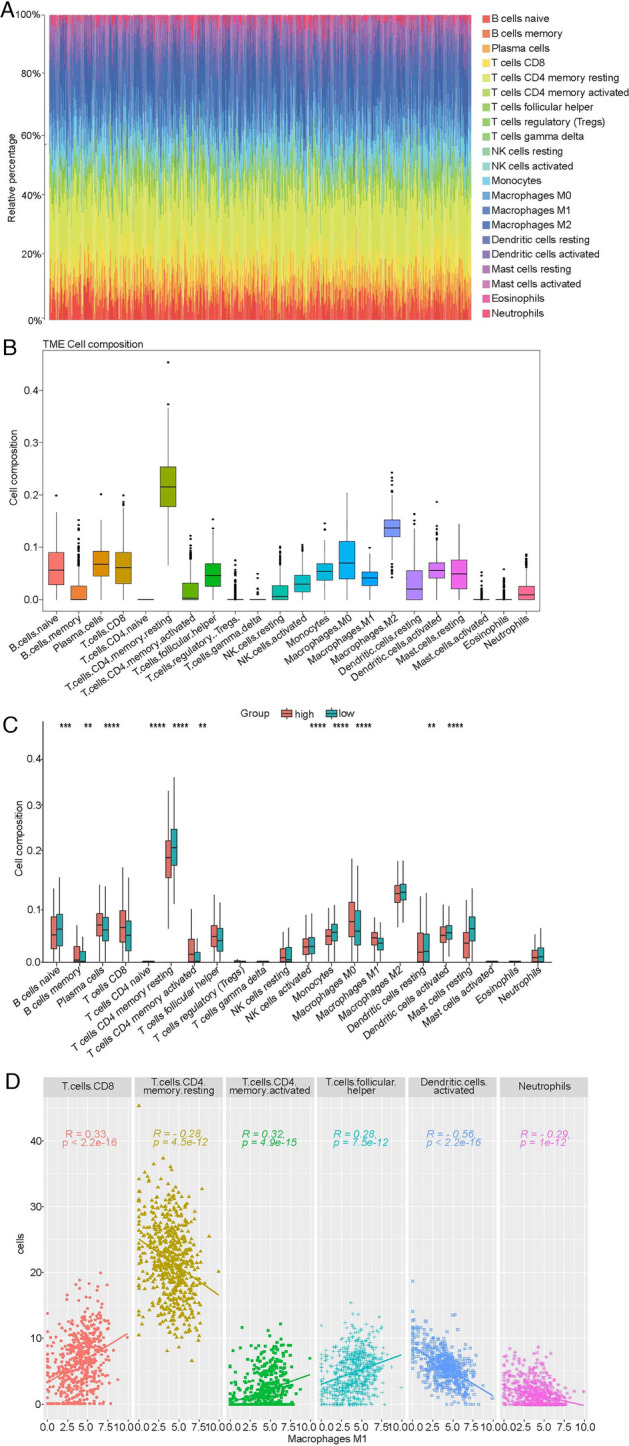
The infiltration of immune cells in high-risk and low-risk patients. (**A**) The relative infiltrating proportion of 22 immune cells in all LUAD samples. (**B**) Relative infiltrating proportion of 22 immune cells in each sample. (**C**) Differentially infiltrated immune cells between high-risk and low-risk LUAD patients. (**D**) The correlation of M1 phagocytes with T cells CD8, T cells CD4 memory resting, T cells CD4 memory activated, T cells follicular helper, dendritic cells activated and neutrophils.

The immune infiltration of 22 immune cells between high-risk group and low-risk group were estimated using CIBERSORT method combined with LM22 feature matrix. The immune infiltrate of immune cells in 527 LUAD patients was shown in Fig. 6A,B. The highest proportion of cells was T.cells. CD4.memory.resting followed by Macrophages.M2 and Macrophages.M0. The variation of proportion in tumor infiltrating immune cells among different patients might represent an intrinsic feature of individual differences. In addition, the infiltration proportion of multiple immune cells was significantly different between high-risk and low-risk groups, and the proportion of M1 phagocytes was significantly higher in the high-risk group compared with the low-risk group (Fig. 6C). These results suggested that the poor prognosis in high-risk group might be correlated with M1 phagocyte proliferation or migration resulting from altered expression of 10 genes. Moreover, we analyzed the correlation among 22 immune cells (Fig. S2) and found that M1 phagocytes were positively associated with the T cells CD8, T cells CD4 memory activated and T cells follicular helper, and were inversely correlated with the T cells CD4 memory resting, dendritic cells activated and neutrophils (Fig. 6D), which was consistent with previous results^22,23^.

## Discussion

In our study, we built a prognostic risk score based on 10 drug resistance related genes by comprehensive bioinformatic analyses to explore its potential prognostic value in LUAD patients. We discovered that the high-risk patients exhibited worse prognoses. The drug resistance related genes could predict the prognosis of LUAD patients.

Recently, with the development of the chemotherapy agents, the treatment of LUAD has achieved great improvement. However, almost all LUAD patients will eventually develop chemoresistance to the chemotherapeutic agents^8,11^, thereby leading to a poor prognosis^24^. In the present study, we identified 13 genes that were positively associated with drug resistance in LUAD patients. Among which 10 optimal genes (PLEK2, TFAP2A, KIF20A, S100P, GDF15, HSPB8, SASH1, WASF3, LAMA3 and TCN1) were screened by LASSO Cox regression analysis, and were used to construct prognosis risk score model of LUAD patients. In the training and validation datasets, patients with high-risk scores had a worse prognosis. In addition, of these 10 genes, the overexpression of PLEK2, TFAP2A, KIF20A, S100P and TCN1 was correlated with poor prognosis of LUAD patients^25–28^. GDF15 could inhibit the proliferation, migration and cell growth, and promote apoptosis of LUAD cells^29^. The overexpression of HSPB8 could increase proliferation and migration in LUAD cells^30^. SASH1 was expressed in a variety of tissues, especially in the breast, lung, thymus, thyroid and spleen. It has been reported that SASH1 was down-regulated in tumors of the breast, lung and thyroid^31^. Inversely, the overexpression of SASH1 could increase cisplatin resistance of NSCLC cells, and reduce cellular proliferation and migration^32,33^. WASF3 regulates the dynamics of the actin cytoskeleton, and it involved in the invasion and metastasis of cancer cells^34^. In NSCLC, the WASF3 was highly expressed^35^, and the higher expression of WASF3 was exhibited a lower five-year survival rate in SCLC patients^35^. In pancreatic cancer, the overexpression of WASF3 could promote the proliferation, migration and invasion of cancer cells by regulating the AKT pathway^36^. In addition, the WASF3 transcription is regulated by multiple factors. Signal Transducer and Activator of Transcription 3 (STAT3) is activated by JAK2 kinase and relocates to the nucleus to bind to its binding site on WASF3, thereby increasing the transcription of WASF3^37^. The MDA-MB-231 cells were induced by ABL kinase inhibitor Gleevec (STI-571) could decrease the phosphorylation level of WASF3^38^. Moreover, some microRNA, such as mir-31, mir-93 and mir-217 could regulate the expression of WASF3 in cancers^39–41^. Therefore, we hypothesized that these 10 genes might affect the LUAD cell invasion, metastasis and proliferation by multiple factors, thereby impacting the prognosis of LUAD patients. Moreover, in cancer cells, the gene mutations, amplifications and deletions could render target protein mutations, and target proteins were unable to bind drugs, leading to drug resistance^42^. Missense mutation could observably impact the function of proteins^43^. Thus, we analyzed the mutation of thee 10 genes in LUAD, and found that the missense mutations of SASH1 and WASF3 were 33% and 29%, respectively, and were the highest among the 10 genes. It has been demonstrated that the missense mutation of SASH1 has been shown to be correlated with genodermatosis, such as dyschromatosis universalis hereditarian^44,45^. To date, 15 and 9 missense mutations have been reported in SASH1 and WASF3 in LUAD patients (Table S5). Therefore, it was reasonable to hypothesize that missense mutations of SASH1 and WASF3 might cause target gene mutations of anticancer drugs in LUAD, thereby causing drug resistance in cancer cells. However, the functional characteristics of most missense mutations in SASH1 and WASF3 have not been clearly characterized in tumors.

GSEA enrichment analysis indicated that a total of 18 pathways were significantly activated in high-risk patients compared with low-risk patients. Among which, some metastasis and immune related pathways were found, such as ECM-receptor interaction and PI3K-Akt signaling pathway. ECM is a crucial component of TME and is involved in the invasion, metastasis and drug resistance of cancer cells^46,47^. Moreover, the excessive deposition of ECM was considered one of the hallmarks of tumors correlated with poor prognosis of patients^48^. Therefore, it is reasonable to observe the ECM-receptor interaction was significantly activated in high-risk patients compared with low-risk patients. In addition, it has been reported that the composition of ECM could impact the polarization of TAM. For example, the ECM (fibronectin-rich) could promote M1 phenotype in macrophages^49^. These evidences suggested that ECM could regulate the immunoreaction in TME by altering the polarization of macrophages. In addition, the PI3K-Akt signaling pathway also plays an important role in the polarization of macrophages. The activated PI3Kγ (subunit of PI3K) could suppress NF-κB and promote C/EBPβ activation by recruiting the serine/threonine kinase Akt. The imbalance between NF-κB and C/EBPβ might lead to formation of M2 phenotype in macrophages^50^. In our present study, the M1 phagocyte was significantly higher in the high-risk group compared with the low-risk group. However, whether M1 phenotype in macrophages was regulated or promoted by ECM-receptor interaction and PI3K-Akt signaling pathway, thus causing poor prognosis of LUAD patients needs to be further investigated. Remarkably, M1 phenotype is involved in the inflammatory response and restrains tumor progression^16^. Dendritic cells, as the professional antigen-presenting cells, can process and present antigens to T cells^51^. Mature dendritic cells could migrate to lymphoid organs to interact with T cells and induce immune responses^52^. Bell et al. found that a large of immature dendritic cells were present in breast carcinoma^53^, and the IL-10, TGF-β and VEGF could inhibit maturation and function of dendritic cells in lung carcinomas^54^. These indicated that TME was able to inhibit the maturation of dendritic cells. In our study, M1 phagocytes were positively associated with the T cells CD8, T cells CD4 memory activated and T cells follicular helper, and were inversely correlated with the T cells CD4 memory resting, dendritic cells activated and neutrophils. In addition, Li et al. have found that many genes, such as TNF, HIF1a, IL-6, IL-1β serve key roles in macrophage polarization^55^. Transcriptional factors, STAT1, IRF3, IRF5 were associated with the activation of M1 phenotype induced by toll-like receptor and STAT6, AKT2, KLF4 were correlated with the polarization of M2 macrophages^56,57^. Considering the role of genes in regulating macrophage polarization, we hypothesized that in the high-risk group, the 10 genes resistance related genes might impede the activation of dendritic cells by influencing the polarization of macrophages, leading to poor prognosis in LUAD patients, which needs to further explore in the future studies.

Although multiple public datasets have been integrated to build the predictive model in this work, there are several limitations in our study. Firstly, candidate genes that were positively associated with drug resistance in LUAD were currently selected. Considering the limited sample size, those genes that were negatively associated with drug resistance might also be meaningful for our analysis. In addition, the mechanisms by which cancer cells gain resistance to anticancer treatments include overexpression of drug efflux proteins or poor expression of drug inflow proteins, alterations in drug direct targets, target mutations, as well as quantitative and qualitative alterations in intracellular drug targets^58,59^. Jin and colleagues have reported that high DSG2 expression could increase the Osimertinib (EGFR tyrosine kinase inhibitor) resistance in LUAD^12^. Yin et al. have found that tumor suppressor genes, such as *BRCA1*, *BRCA2* and *MLH1*, involved in the modulating the drug resistance in ovarian cancer via regulating DNA damage-related and apoptosis related signaling pathways^60^. Rsf-1 ectopic expression dramatically increased paclitaxel resistance in ovarian cancer cells^61^. These evidences indicated that abnormal target gene expression was associated with drug resistance in tumors. In the present study, we revealed PLEK2, TFAP2A, KIF20A, S100P, GDF15, HSPB8, SASH1, WASF3, LAMA3 and TCN1 were target genes of multiple drugs (Fig. S3), and the expression of these 10 genes was prominently positively related to drug resistance in LUAD. Therefore, abnormal expression of these 10 genes might be related to drug resistance in LUAD. However, we are still required to validate our results further through biological experiments in vitro or in vivo. Moreover, whether tumor resistance was associated with drug target alterations and post-translational modifications requires further investigation in LUAD. Moreover, the single gene expression in risk score model was not investigated in LUAD patients, and the correlation between gene expression and the prognosis of LUAD patients was not explored.

## Conclusions

In conclusion, we identified 10 novel drug resistance related genes (PLEK2, TFAP2A, KIF20A, S100P, GDF15, HSPB8, SASH1, WASF3, LAMA3 and TCN1) in LUAD. The risk score model of LUAD constructed with these 10 genes could reliably predict the prognosis of LUAD patients, The high-risk patients had a worse prognosis. Our research could provide more information for understanding the link between the drug resistance genes and TME in LUAD patients, and could contribute to new targets for drug development in the future.

## Supplementary Information

Supplementary Table S1.

Supplementary Table S2.

Supplementary Table S3.

Supplementary Table S4.

Supplementary Table S5.

Supplementary Figure S1.

Supplementary Figure S2.

Supplementary Figure S3.

## Data Availability

The datasets analyzed during the current study are available in The Cancer Genome Atlas database (TCGA, https://tcga-data.nci.nih.gov/tcga/) and the Gene Expression Omnibus database (GEO, https://www.ncbi.nlm.nih.gov/geo/) [GSE43458, GSE32863, GSE42127].

## Abbreviations

LUAD: Lung adenocarcinoma
NSCLC: Non-small lung cancer
TCGA: The Cancer Genome Atlas
SCLC: Small cell lung cancer
TAMs: Tumor-associated macrophages
TME: Tumor microenvironment
GEO: Gene Expression Omnibus
DEGs: Differentially expressed genes
GO: Gene ontology
KEGG: Kyoto Encyclopedia of Genes and Genomes
LASSO: Least absolute shrinkage selection operator
CNV: Copy number variation
GSEA: Gene set enrichment analysis
AUCs: Area under curves
ECM: Extracellular matrix
STAT3: Signal Transducer and Activator of Transcription 3
