# Supplementary material for: Drug resistance related genes in lung adenocarcinoma predict patient prognosis and influence the tumor microenvironment
Source: Sci Rep. 2023 Jun 15;13:9682. doi: 10.1038/s41598-023-35743-y (PMC10272185; doi:10.1038/s41598-023-35743-y)
Supplement: Supplementary file 6 — Supplementary Figure S1. [file 41598_2023_35743_MOESM6_ESM.docx]

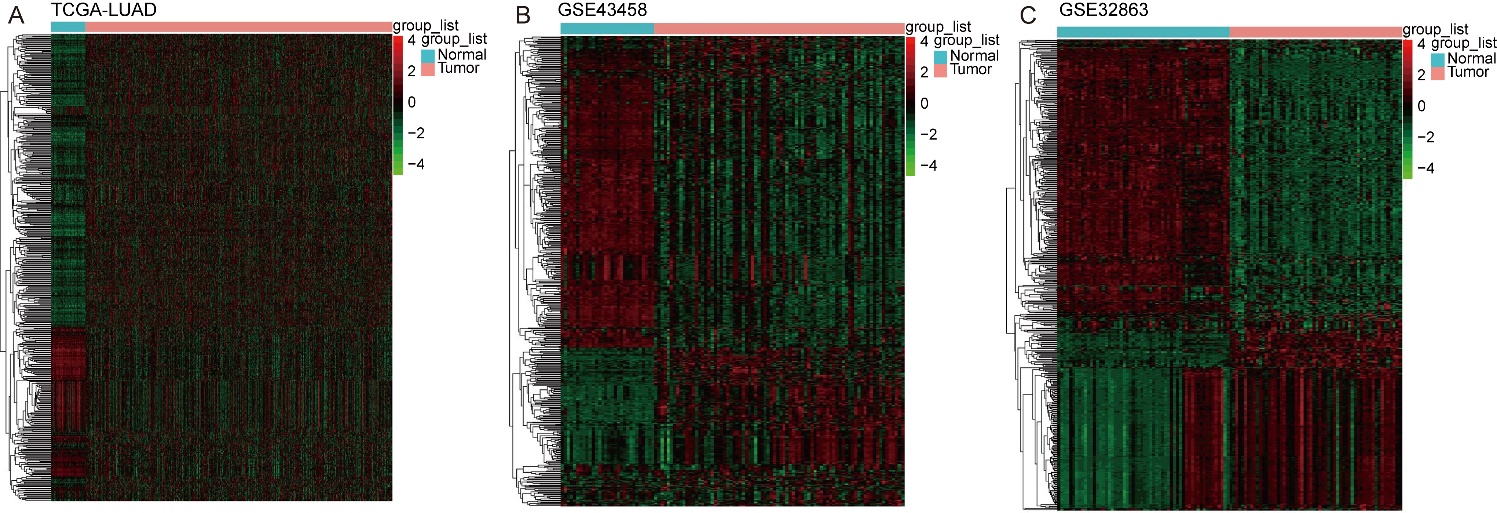


Figure S1 The heatmap of DEGs between cancer group and paracancerous sample in TCGA-LUAD dataset. (A) GSE43458 dataset (B) and GSE32863 dataset (C) The heatmap was plotted using the pheatmap (Version 1.0.12, https://cran.r-project.org/web/packages/pheatmap/index.html).
