# Supplementary figures and images for: Drug resistance related genes in lung adenocarcinoma predict patient prognosis and influence the tumor microenvironment
Source: Sci Rep. 2023 Jun 15;13:9682. doi: 10.1038/s41598-023-35743-y (PMC10272185; doi:10.1038/s41598-023-35743-y)

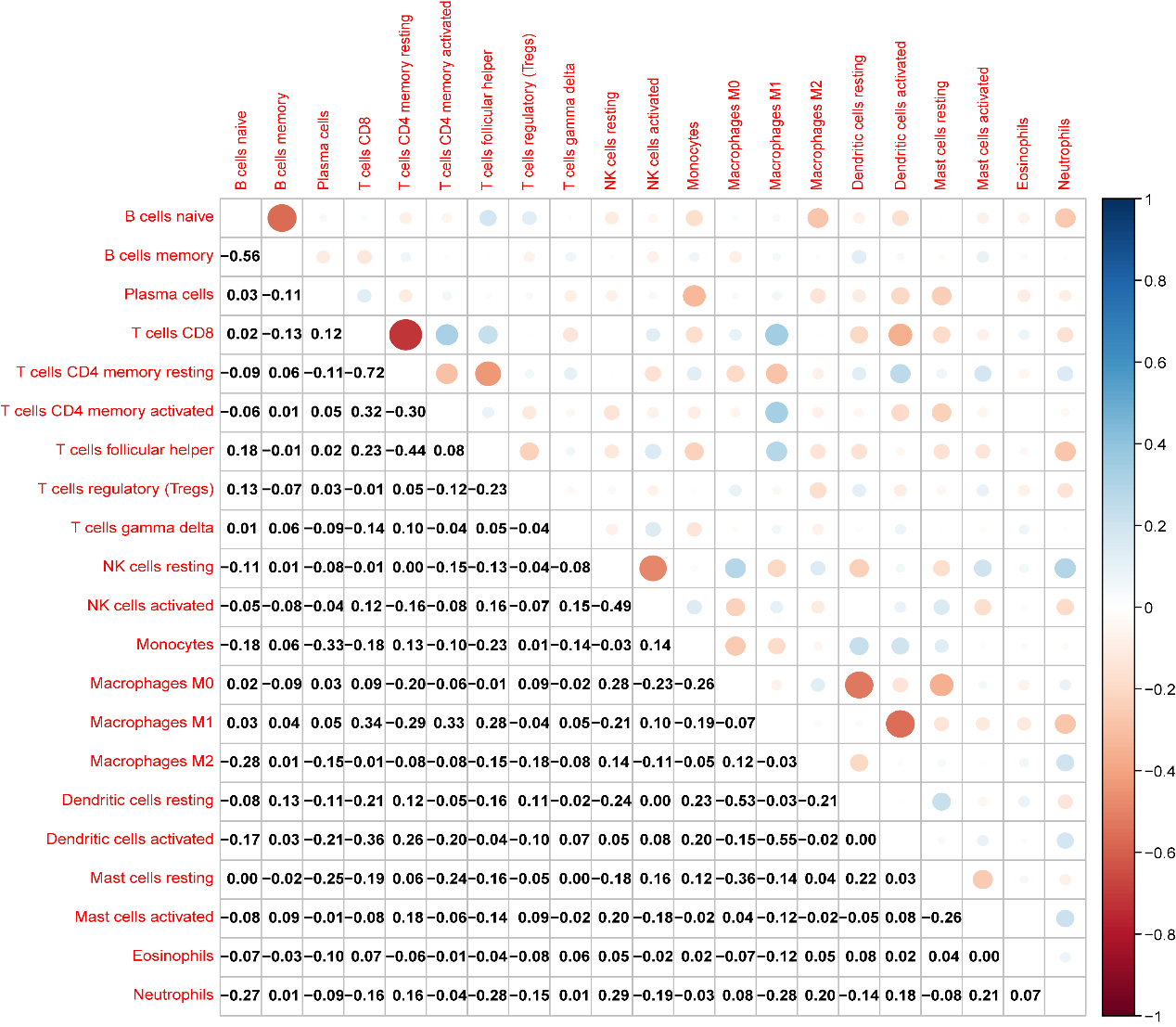


Figure S2 The correlation between immune cells infiltration

Supplement: Supplementary file 7 — Supplementary Figure S2. [file 41598_2023_35743_MOESM7_ESM.docx]

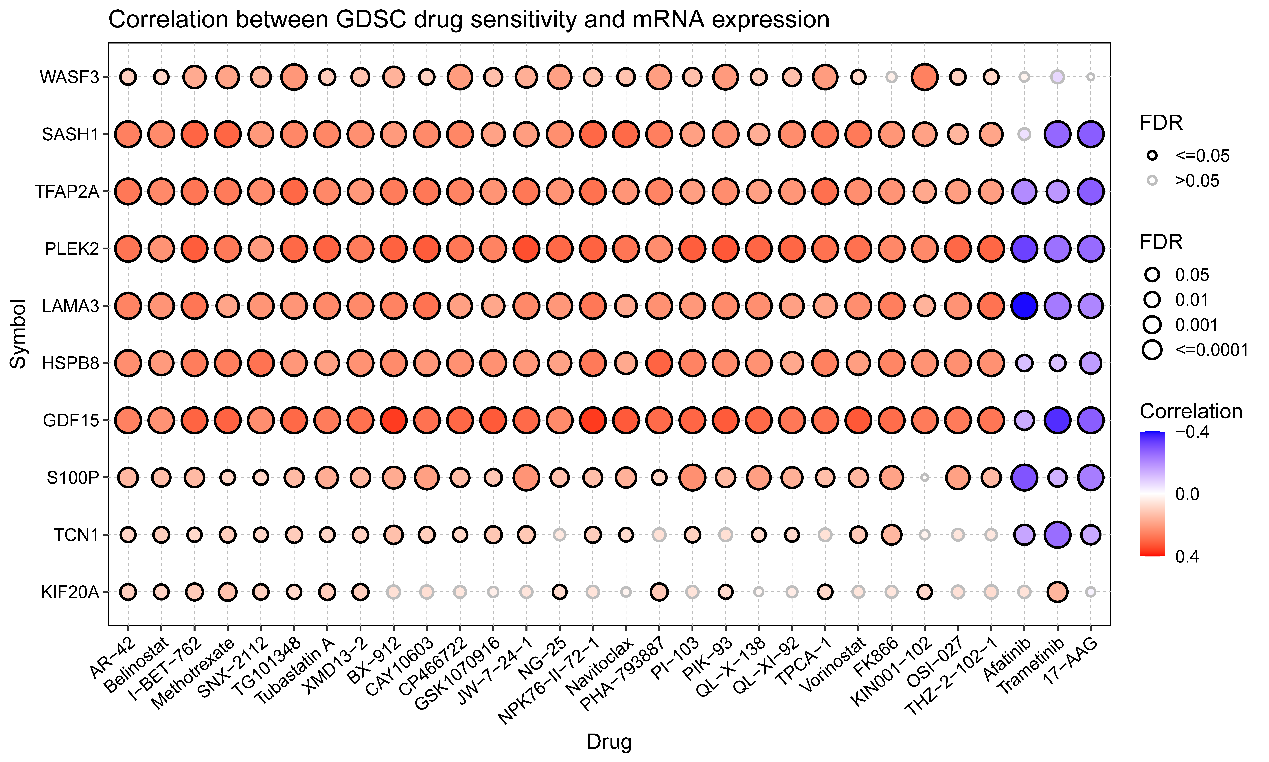


Figure S3 The PLEK2, TFAP2A, KIF20A, S100P, GDF15, HSPB8, SASH1, WASF3, LAMA3 and TCN1 were targets of multiple drugs

Supplement: Supplementary file 8 — Supplementary Figure S3. [file 41598_2023_35743_MOESM8_ESM.docx]
